# Phytochemical diversity within and among Sardinian populations of the endemic *Teucrium marum* L. (Lamiaceae) is determined by ecological factors

**DOI:** 10.1016/j.heliyon.2023.e17728

**Published:** 2023-06-29

**Authors:** Alfredo Maccioni, Silvia Macis, Marc Gibernau, Emmanuele Farris

**Affiliations:** a Dept. of Chemical, Physical, Mathematical and Natural Sciences, University of Sassari, Via Piandanna, 4, 07100 Sassari, Sardinia, Italy; b Dept. of Life and Environmental Sciences, University of Cagliari, Viale S. Ignazio, 13, 09123 Cagliari, Sardinia, Italy; c Laboratory of Sciences for the Environment (UMR 6134), CNRS – University of Corsica, Vignola – Route des Sanguinaires, 20000 Ajaccio, France; d NBFC, National Biodiversity Future Center, Palermo 90133, Italy

**Keywords:** Cat thyme, dolichodial, *(E)*-*β*-caryophyllene, Elevation gradient, Essential oils, GC and GC–MS analysis

## Abstract

Wild aromatic plants present high intra- and inter-population chemical polymorphisms which are of great ecological and economic interest; however, the factors influencing the phytochemical diversity of aromatic plants remain still unexplored for many species. Here, *Teucrium marum* L. (Lamiaceae) has been studied, a plant endemic to the western Mediterranean islands, very well-known from the phytochemical point of view but scarcely investigated regarding the ecological factors that influence its phytochemical diversity within and among populations. The specific aims were to: 1) define the chemical composition variability of its essential oils; 2) determine its inter- and intra-population chemical variability; and 3) evaluate whether the elevation, climatic factors and/or the soil substrate determined the phytochemical variability of *T. marum* along a gradient from coastal to mountain wild populations on the island of Sardinia (Italy). Fresh *T. marum* aerial parts were randomly collected from ten individuals in six different localities in Sardinia: three coastal and three mountainous. Dried leaf samples were hydrodistillated using a classical Clevenger apparatus to obtain the corresponding essential oils. The composition of each essential oil was chemically characterised and analyzed by gas chromatography coupled to mass spectrometry. Ninety compounds were identified: among the others, the two compounds that mainly characterised the essential oils of the studied populations, dolichodial and *(E)*-*β*-caryophyllene, are of great economic interest. Statistical analyses showed significant differences in phytochemical essential oil composition among and within the studied populations, which clustered following a geographical pattern rather than a simple climatic or edaphic grouping. Taken together the results here shown shed light on the environmental and geographical conditions that determine the chemical variability of essential oils in *T. marum*, highlighting a clear coastal vs mountain clustering, which has an ecological and economic relevance, especially for the potential utilization of dolichodial as an environmental-friendly insecticide.

## Highlights

• Cat thyme showed significant chemical variability among and within populations.
• dolichodial and *(E)-β-*caryophyllene were the main compounds.
• Ecological factors determine chemical variability of essential oils in cat thyme.
• Several compounds, and especially dolichodial, are of great economic interest.
• dolichodial showed different abundance among coastal and mountain populations.

## 1 Introduction

Plant secondary metabolites, now designated as specialized metabolites, like terpenes and phenolic compounds, are known to have multiple ecological roles, notably to protect plants against herbivores, pathogens, and abiotic stresses, but also to modulate plant–plant or plant–animal interactions [[1], [2], [3], [4], [5], [6], [7]]. Their production depends on the physical and chemical properties of the ecosystem, like precipitation, temperature, and edaphic conditions, but also elevation [[1], [2], [3], [4],^8^,^9^]. Therefore, wild aromatic plants present high chemical polymorphisms in response to the contrasted environmental conditions and geographical distances of their different populations, leading to an important intra- and inter-population chemical variability [[8], [9], [10], [11], [12], [13]]. In the same way, the variability of the essential oil (EO) composition of the natural populations is related also to various environmental, geographical, and genetic factors, which influence, in combination or independently, the essential oils (EOs) quantitative and qualitative composition and properties [[1], [2], [3],^9^,[14], [15], [16], [17], [18], [19], [20], [21], [22], [23], [24], [25]]. Essential oils from Medicinal and Aromatic Plants (MAPs) have been considered of special economic interest, as in the pharmaceutical, medical, and agricultural fields, by virtue of their biologically active compounds [5,8,[26], [27], [28], [29], [30], [31]]. Of the 153 genera (from 49 families) of vascular plants that accumulate EOs, 90 occur in Mediterranean-type ecosystems [3], which are characterised by a climate regime with mild, wet winters, and warm, dry summers and with 90% or more of the annual precipitation typically occurring in the 6 months around winter [32]. The Mediterranean basin, one of the five Mediterranean areas of the world, has a high geographical and ecological heterogeneity, which has originated and continues to cause original patterns of vascular plant evolution at the population, community and landscape levels [3]. For this reason, this basin has recently been defined as a complex, multi-hierarchical system of islands-within-islands [33]. In this context, the combination of several evolutionary factors, such as richness in microhabitats and geographical isolation over long periods, has promoted further diversification among and within species, giving rise to a huge contingent of Mediterranean-exclusive (endemic) species particularly abundant in harsh environments [34], where secondary compounds evolved mainly as a defence against herbivores and insects [3,5]. Even, however, if there are some well documented studies showing evidence of differentiation of morphologic, genetic, and phytochemical aspects among and within populations of endemic aromatic plants growing in harsh Mediterranean environments [19,35,36], the ecological factors influencing these patterns still need to be revealed for many aromatic species of ecological and economic interest.

In this study, *Teucrium marum* L., commonly known as cat thyme and belonging to the Lamiaceae family, was chosen as a model species. In fact, this western Mediterranean endemic dwarf shrub is well known for its phytochemical properties, but its phytochemical diversity as influenced by ecological factors within and among populations has not been thoroughly studied. Previous research on the phytochemical composition of its EO has been done in Sardinia and Corsica [5,30,[37], [38], [39]], but many studies from Sardinia were limited to one or a few populations. Meanwhile, the main study in Corsica was focused primarily on analytical procedures and cannot provide ecological insights [40].

Therefore, the general purpose of this paper is to investigate the hypothesis that ecological factors can influence the intra- and inter-population phytochemical diversity of this species.

The specific aims were to: 1) define the chemical composition variability of its EOs, especially focusing on compounds of economic interest; 2) determine its inter- and intra-population chemical variability; and 3) evaluate whether the elevation gradient (as a proxy of climatic factors), climatic factors and/or soil substrate determine the phytochemical variability of *T. marum* from different coastal and mountain wild populations on the island of Sardinia (Italy).

## 2 Materials and methods

### 2.1 Study area

Sardinia, the second-largest island of the Mediterranean Basin, with an area of 24,089 km^2^, represents one of the 10–11 Mediterranean biodiversity hotspots [[41], [42], [43], [44], [45], [46]]. Its climate is characterised by a Mediterranean pluvi-seasonal oceanic macrobioclimate and 43 detailed iso-bioclimates [47,48]. Its flora comprise ca. 2300 native vascular plant taxa, of which ca. 15% are endemic to the Sardinian–Corsican biogeographic province and the surrounding Tyrrhenian areas, therefore highlighting the originality of the islands’ genetic and geological history [41,[49], [50], [51], [52]].

In this context, *T. marum* is one of the most characteristic species of the dwarf shrub vegetation (garrigues) often derived by human-induced degradation of pristine evergreen forests through fire, grazing, and/or clearing. This endemic chamaephyte has been chosen as a guide species to name the phytosociologic alliance *Teucrion mari* Gamisans & Muracciole 1984 [53], including several Sardinian–Corsican communities dominated by dwarf perennial plants.

**Fig. 1:**
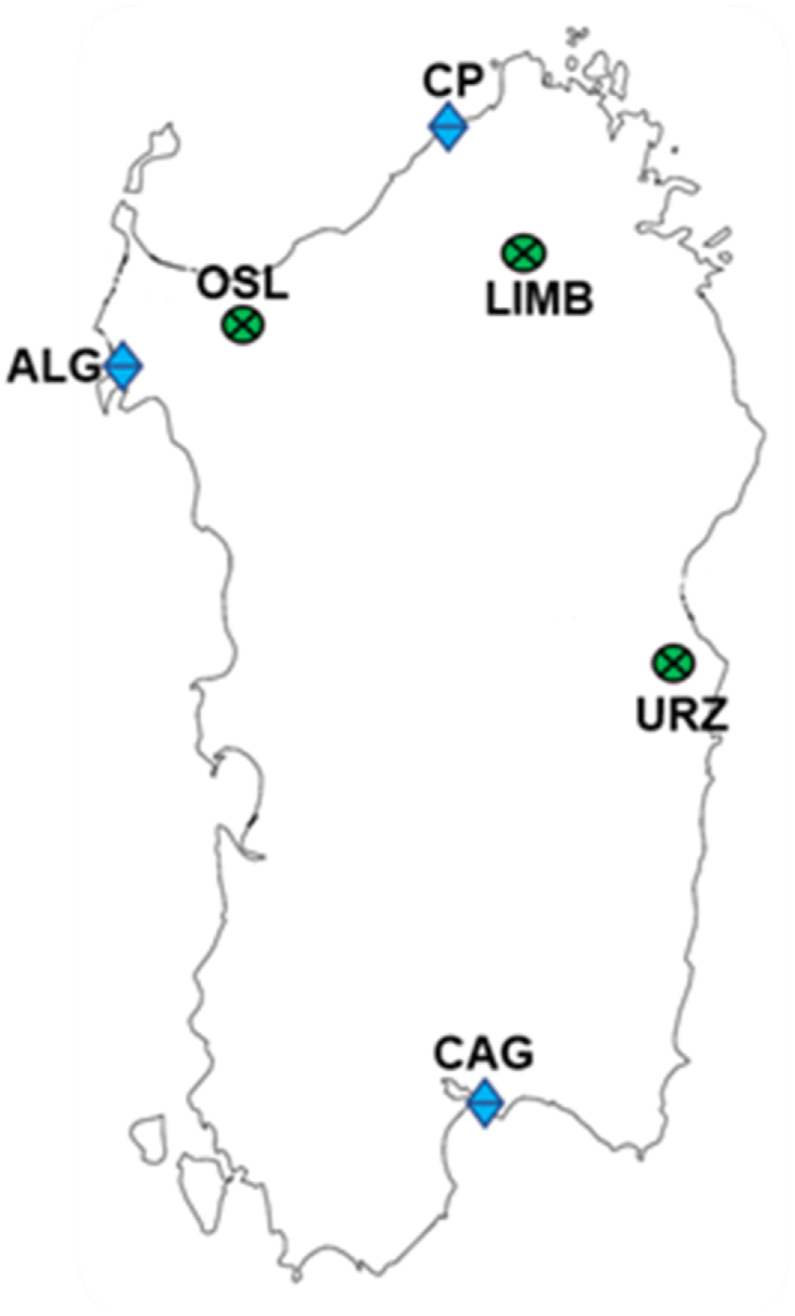
Sampled Sardinian populations of *Teucrium marum* L. Costal localities: CAG (Capo S. Elia – Cagliari, CA), ALG (Lazzaretto – Alghero, SS), and CP (Costa Paradiso - Trinità d'Agultu e Vignola, SS). Mountainous localities: LIMB (Mt. Limbara - Tempio Pausania, SS), URZ (Genna Silana – Urzulei, NU), and OSL (Mt. Tuffudesu – Osilo, SS).

Fresh *T. marum* aerial parts were collected during their flowering period (May–June 2022) in six different localities in Sardinia: three coastal and three mountainous (Table 1, Fig. 1). For each station, ten individual plants were randomly sampled at a minimum distance of 20 m between samples in order to avoid sampling siblings.

**Table 1:** Wild Sardinian populations of *Teucrium marum* L. data.

Population code	Site name and Municipality (Province)	WGS84 coordinates	Elevation (m a.s.l.)	Soil substrate	Bioclimate^a^
CAG	Capo S. Elia – Cagliari (CA)	39.1855475 N 9.1475736 E	41	Carbonate	2 – Lower thermo-Mediterranean, lower dry, strong euoceanic
ALG	Lazzaretto – Alghero (SS)	40.5839220 N 8.2482990 E	44	Dolostone	8 – Upper thermo-Mediterranean, upper dry, semi-hyperoceanic
CP	Costa Paradiso - Trinità d'Agultu e Vignola (SS)	41.0529210 N 8.9609560 E	174	Granites	9 – Upper thermo-Mediterranean, upper dry, strong euoceanic
LIMB	Mt. Limbara - Tempio Pausania (SS)	40.8485980 N 9.1646290 E	1263	Granites	40 – Lower supratemperate, lower humid, weak semi-continental
OSL	Mt. Tuffudesu - Osilo (SS)	40.7348590 N 8.6807020 E	704	Andesitic	28 – Upper meso-Mediterranean, upper subhumid, weak euoceanic
URZ	Genna Silana – Urzulei (NU)	40.1591710 N 9.5064150 E	1012	Carbonate	35 – Lower supra-Mediterranean, lower humid, weak semi-continental

a Numbers and bioclimate diagnosis refer to [48].

### 2.2 Study species

The highest diversification of Mediterranean endemic species rich in secondary compounds occurs in the families Apiaceae, Asteraceae and Lamiaceae [3,31]. Lamiaceae is a family of aromatic plants, including the genus *Teucrium* L. (germander), which includes 300 species distributed in Europe, North Africa, and temperate areas of Asia but which is mainly concentrated in the Mediterranean region [38,54]. The genus *Teucrium* is characterised by herbs or shrubs, with tubular or campanulate calyx, two-lipped or actinomorphic and five-toothed, with the upper teeth equal to or larger than the lower. Corolla has one five-lobed lip, a tube without a ring of hairs inside, often included in the calyx. Nutlets are smooth or reticulate.

The studied species, *Teucrium marum* L., is a perennial aromatic suffruticose chamaephyte characteristic of arid and stony environments (e.g., garrigues), from 0 to 1200 (rarely up to 1700) m a.s.l., endemic to the islands of Sardinia, Capraia, Montecristo, and Gorgona in Italy, Corsica, Hyères and Murter in France, and the Balearic archipelago in Spain. Cat thyme is an aromatic and medical plant used in traditional medicine, with diuretic, nervine, stimulant, stomachic, tonic, antibacterial, anti-inflammatory, antipyretic and cicatrising properties, astringent, and used to control haemorrhages [5,30,38,55]. The biological properties of a single EO of cat thyme are determined, generally, by its main components [38,56].

### 2.3 Plant material and essential oil

The methodology followed the standard analytical protocol, described in accordance with the European Pharmacopoeia, and followed for *T. marum* by Ref. [5].

To avoid the singularity of the volatile compounds being influenced by the sampling period and different extraction methods, compared with other reports [5,30,[38], [39], [40],^57^,^58^], field sampling was carried out over a short period of time during the flowering stage, and a standard extraction method for EOs was used [5].

For each harvested individual, the first twenty cm of canopy (leaves and inflorescent parts at the same phenological state) were collected. Samples were dried in a heating oven 100% ventilated, set to 40 °C for 48 h in order to avoid any degradation or loss of EO.

Dried leaves of each sampled plant were hydrodistillated separately and introduced into a 2 L wide-neck flask reaction (QFR2LF, Quickfit®, Fisher Scientific, Pittsburgh, Pennsylvania, USA). One liter of distilled water was added and heated for 2 h using a heating mantle (EM2000/CE. Electrothermal®, Vernon Hills, Illinois, USA). The EO was collected from each sampled plant using a classical Clevenger apparatus, cooled to 6 °C using a refrigerated fluid (a mix of glycol and water) and moved by a Minichiller® (C_20_. Huber®, Freiburg, Germany). The EOs were then diluted in 0.5 mL of pentane in the Clevenger due to the low EO volume produced by some samples, and transferred to a Gas Chromatography (GC), vial to which 5 mL of pentane was added before sealing. The GC vials were sealed and stored at 4 °C before further analysis.

### 2.4 GC and GC–MS analysis

Gas Chromatography (GC), coupled with Mass Spectrometry (MS), was used to analyse EOs in this study: this is widely used method in EO studies is also an important technique for the quality evaluation and control of natural products [59,60].

Gas Chromatography analyses, a widely used method in EO studies, were performed on a Perkin Elmer Clarus 500 gas chromatograph (FID, Perkin Elmer, Courtaboeuf, France), equipped with 2 fused silica gel capillary columns (50 m. 0.22 mm id. film thickness 0.25 μm): BP^−1^ (polydimethylsiloxane) and BP^−20^ (polyethylene glycol). The oven temperature was programmed from 60 to 220 °C at 2 °C/min and then held isothermally at 220 °C for 20 min. The injector temperature was 250 °C, detector temperature 250 °C, carrier gas hydrogen (1.0 mL/min) and split of 1/60.

The EOs were also analyzed with a PerkinElmer gas chromatography coupled with mass spectrometry (GC/MS), TurboMass detector (quadrupole, PerkinElmer, Courtaboeuf, France) directly coupled with a PerkinElmer Auto system XL, equipped with a fused silica gel capillary column (50 m. 0.22 mm id. film thickness 0.25 μm) (BP^−1^ polydimethylsiloxane). The analyses were performed with the following parameters. Helium was the carrier gas at 0.8 mL/min. The injection volume was 0.5 μL, with a split of 1/75 at an injector temperature of 250 °C. The oven temperature was programmed from 60 to 220 °C with a 2 °C/min increase and finally held iso-thermally (20 min). The ion source temperature was 250 °C, with an ionisation energy of 70 eV. The electron ionisation mass spectra were acquired over the mass range of 40–400 Da.

The GC retention indices (RIs) of each compound were determined on both polar and apolar columns, relative to the retention times of a series of n-alkanes (C_7_–C_28_) with linear interpolation (‘Target Compounds’ software, PerkinElmer). The compounds were first identified by comparing their two RIs with those of authentic compounds and literature data. These identifications were also confirmed by mass spectral comparison using a reverse match factor >900 with commercial reference libraries (FFNSC2, Nist11W9, Perkin_ff2013, Wiley8) (Fig. S1). For each compound, the mean relative amount (%) per population, obtained from ten sampled specimens.

### 2.5 Data analysis

Statistical analyses were performed with Past 4.11 statistical software [61]. In accordance to Hervé et al. [62] raw data with compound percentages were clr-transformed (Center logratio) prior to analysis to normalise the weight of all the compounds. Individual phytochemical compositions were visualised in a Principal Component Analysis (PCA) with triplot for environmental factor, the compounds differentiating the populations were determined with a Similarity Percentage (SIMPER) analysis, finally the statistical population phytochemical differences were tested with an Analysis of Similarities (ANOSIM), and the effect of environmental factors (elevation and soil substrate) by a Permutational Multivariate Analysis of Variance (PERMANOVA with 9999 replicates for each). According to ter Braak [63], who suggested Canonical Correspondence Analysis (CCA) as the technique that best explains the variation in an abundance matrix with a combination of environmental variables, we also examined the relationship between compounds founded in our populations, aggregated as groups, and identified the most important environmental variables associated with those compounds.

In the PCA, climatic factors (extracted from Refs. [47,48]) and substrate (extracted from Ref. [64]) were plotted as supplementary variables to visualise any correlation between phytochemical composition and environmental data, such as elevation, mean annual temperature, maximum temperature of the warmest month, minimum temperature of the coldest month, mean annual precipitation, precipitation of the wettest month, and precipitation of the driest month.

SIMPER was applied to assess which compounds were primarily responsible for the observed differences between populations [65]. The Euclidean similarity measure was used for the SIMPER analysis. The overall significance of the difference was assessed by a one-way ANOSIM, and among population differences with a post-hoc test of multiple pairwise-population comparisons using Bonferroni-corrected *p* values. The factor effect (elevation, soil substrate (see Table 1) and their interaction) was tested with a two one-way PERMANOVA. Differences with *p* ≤ 0.05 between groups were considered significant.

## 3 Results and discussion

### 3.1 Phytochemical analysis

Overall, 90 compounds were identified (Table 2) in the EOs of *T. marum* from six different wild Sardinian populations.

**Table 2:** List of the components identified in essential oils obtained from six different wild Sardinian populations of *Teucrium marum* L. For each compound, the mean relative amount per population from ten specimens ± standard error is reported. CAG (Capo S. Elia – Cagliari, CA), ALG (Lazzaretto – Alghero, SS), CP (Costa Paradiso – Trinità d'Agultu e Vignola, SS), LIMB (Limbara – Tempio Pausania, SS), OSL (Osilo, SS), and URZ (Genna Silana – Urzulei, NU).

Compound name	RI^b^ (ap.)	RI^c^ (p.)	CAG (%)	ALG (%)	CP (%)	LIMB (%)	OSL (%)	URZ (%)
2-Methylbutanol	718	1199	0.04 ± 0.03	0.07 ± 0.02	0.06 ± 0.02	–	0.25 ± 0.05	0.08 ± 0.02
Hexanal	773	1078	0.12 ± 0.04	0.13 ± 0.08	0.72 ± 0.18	0.26 ± 0.03	0.24 ± 0.04	0.29 ± 0.05
2-Hexenal	824	1218	0.07 ± 0.03	0.03 ± 0.02	0.01 ± 0.01	0.16 ± 0.03	0.14 ± 0.03	0.21 ± 0.02
Hexanol	846	1345	0.02 ± 0.01	–	0.25 ± 0.06	0.08 ± 0.03	0.17 ± 0.04	0.14 ± 0.04
2-Methylpropyl-propionate	847	1336	–	0.15 ± 0.11	–	–	–	–
3-Methylbutyl acetate	857	1117	0.59 ± 0.17	1.00 ± 0.20	1.02 ± 0.54	0.21 ± 0.06	0.78 ± 0.13	1.36 ± 0.21
3-Heptenol	930	1584	–	–	–	–	–	0.08 ± 0.03
2-Methylpropyl-butanoate	936	1266	–	0.40 ± 0.08	0.19 ± 0.05	0.15 ± 0.05	0.62 ± 0.13	0.04 ± 0.02
3-Methylbutyl-propanoate	952	1183	–	0.72 ± 0.20	0.19 ± 0.07	0.18 ± 0.06	0.89 ± 0.17	0.04 ± 0.01
6-Methyl-5-hepten-2-one	960	1334	1.15 ± 0.26	0.82 ± 0.22	1.73 ± 0.41	0.95 ± 0.13	0.68 ± 0.10	1.14 ± 0.12
Butyl-butyrate	975	1265	–	0.02 ± 0.01	0.03 ± 0.02	0.02 ± 0.01	0.04 ± 0.02	–
2-Pentyl-furan	977	1233	–	–	–	0.03 ± 0.01	0.03 ± 0.01	0.02 ± 0.01
Myrcene	981	1152	0.07 ± 0.07	0.01 ± 0.01	–	–	0.02 ± 0.01	0.01 ± 0.01
*(Z)-*3-Hexenyl acetate	985	1314	–	0.06 ± 0.02	0.01 ± 0.01	0.05 ± 0.02	0.05 ± 0.01	0.10 ± 0.01
2-Methyl-propyl-2-methylbutyrate	988	1265	–	0.30 ± 0.11	0.12 ± 0.03	0.05 ± 0.02	0.33 ± 0.05	0.02 ± 0.01
Hexyl acetate	993	1268	0.35 ± 0.10	1.19 ± 0.90	4.54 ± 1.64	1.66 ± 0.26	0.59 ± 0.09	1.52 ± 0.34
4-Methylanisole	999	1436	0.04 ± 0.01	0.06 ± 0.03	0.01 ± 0.01	0.02 ± 0.01	–	–
3-Methylbutyl-2-methylpropanoate	1001	1280	–	0.18 ± 0.06	0.05 ± 0.02	0.02 ± 0.01	0.19 ± 0.03	–
Limonene	1021	1203	6.16 ± 6.14	–	–	–	–	–
(*E*)-*β*-Ocimene	1025	1230	0.45 ± 0.34	0.06 ± 0.02	0.02 ± 0.01	0.02 ± 0.01	0.10 ± 0.01	0.01 ± 0.01
2-Methylbutyl-butyrate	1040	1258	0.04 ± 0.04	5.76 ± 0.99	6.56 ± 2.64	2.55 ± 0.55	9.54 ± 1.40	1.25 ± 0.52
Octanol	1070	1449	0.03 ± 0.01	–	0.01 ± 0.01	0.01 ± 0.01	0.02 ± 0.01	0.02 ± 0.01
Nonanal	1081	1424	0.01 ± 0.01	–	0.02 ± 0.01	0.11 ± 0.02	0.18 ± 0.02	0.07 ± 0.01
Linalool	1082	1542	0.40 ± 0.08	0.53 ± 0.11	0.62 ± 0.20	0.17 ± 0.05	2.07 ± 0.25	0.71 ± 0.09
Hexyl propanoate	1086	1378	–	0.40 ± 0.30	1.44 ± 0.47	0.89 ± 0.25	0.57 ± 0.13	0.11 ± 0.07
2-Methylbutyl-2-methylbutyrate	1088	1415	–	5.95 ± 1.68	5.08 ± 2.03	1.29 ± 0.21	7.58 ± 0.90	0.61 ± 0.21
Octen-3-yl acetate	1092	1374	0.37 ± 0.10	0.35 ± 0.12	1.81 ± 0.70	0.86 ± 0.14	0.37 ± 0.07	1.11 ± 0.15
(*Z*)-Tagetone	1123	1527	–	0.01 ± 0.01	–	0.01 ± 0.01	0.05 ± 0.02	0.03 ± 0.01
Citronellal	1130	1475	1.73 ± 0.54	0.70 ± 0.35	0.39 ± 0.10	1.12 ± 0.37	1.14 ± 0.24	0.49 ± 0.14
1.4-Dimethoxy-benzene	1131	1733	0.09 ± 0.03	0.17 ± 0.06	0.21 ± 0.08	0.19 ± 0.03	0.10 ± 0.01	0.06 ± 0.03
3-Methylbutyl-pentanoate	1137	1493	–	0.03 ± 0.02	0.02 ± 0.01	0.01 ± 0.01	0.14 ± 0.03	0.01 ± 0.01
Nonanol	1157	1671	–	0.01 ± 0.01	0.01 ± 0.01	0.01 ± 0.01	0.02 ± 0.01	0.01 ± 0.01
(*E*)-3-Hexenyl-butyrate	1165	1662	–	0.05 ± 0.02	0.02 ± 0.01	0.09 ± 0.03	0.15 ± 0.03	0.11 ± 0.02
*α*-Terpineol	1169	1674	–	0.01 ± 0.01	–	–	0.09 ± 0.03	0.03 ± 0.01
Estragole + hexyl butyrate	1172	1664	1.19 ± 0.28	4.51 ± 0.84	5.92 ± 1.06	5.45 ± 0.54	5.15 ± 0.53	5.40 ± 1.09
Isopentyl-hexanoate	1199	1234	0.04 ± 0.02	–	0.01 ± 0,01	–	0.14 ± 0.03	0.05 ± 0.02
3-4-Dimethoxytoluene	1203	1800	0.20 ± 0.13	0.20 ± 0.12	0.02 ± 0.01	0.02 ± 0.01	0.13 ± 0.06	0.14 ± 0.03
Citronellol	1207	1763	0.17 ± 0.08	0.04 ± 0.04	0.01 ± 0.01	0.40 ± 0.16	0.24 ± 0.11	0.08 ± 0.05
Neral	1211	1674	–	0.12 ± 0.06	0.06 ± 0.03	0.04 ± 0.03	0.34 ± 0.08	0.52 ± 0.18
(*Z*)-3-Hexenyl-2-methylbutanoate	1213	1427	–	0.04 ± 0.03	0.02 ± 0.01	0.02 ± 0.01	0.13 ± 0.02	0.07 ± 0.02
Hexyl-2-methylbutanoate	1220	1416	–	0.33 ± 0.27	2.19 ± 0.61	1.60 ± 0.19	1.03 ± 0.20	0.86 ± 0.27
Phenylethyl acetate	1222	1809	0.18 ± 0.07	0.38 ± 0.11	0.45 ± 0.10	0.13 ± 0.04	0.11 ± 0.02	0.86 ± 0.13
Geraniol	1232	1840	0.04 ± 0.02	0.05 ± 0.02	0.03 ± 0.01	0.02 ± 0.01	0.70 ± 0.12	0.15 ± 0.03
2-Methylbutyl-hexanoate	1234	1475	0.02 ± 0.02	0.09 ± 0.05	0.07 ± 0.03	0.08 ± 0.05	0.44 ± 0.08	0.28 ± 0.06
Geranial	1239	1724	0.45 ± 0.19	0.29 ± 0.13	0.15 ± 0.07	0.15 ± 0.07	0.75 ± 0.17	0.99 ± 0.33
Dolichodial	1253	1920	15.65 ± 4.52	34.38 ± 7.87	11.25 ± 2.45	0.51 ± 0.42	5.32 ± 1.69	5.77 ± 1.30
Epidolichodial	1264	1937	2.54 ± 1.12	4.76 ± 0.99	1.15 ± 0.33	0.02 ± 0.02	0.83 ± 0.30	1.12 ± 0.29
2-Undecanone	1270	1592	0.20 ± 0.08	0.07 ± 0.03	0.22 ± 0.08	0.05 ± 0.02	0.20 ± 0.04	0.31 ± 0.04
6-Undecanol	1308	1807	0.01 ± 0.01	–	–	–	0.03 ± 0.01	0.01 ± 0.01
Benzyl-butyrate	1312	1819	–	0.03 ± 0.01	–	0.01 ± 0.01	0.02 ± 0.01	0.01 ± 0.01
Eugenol	1325	2158	0.06 ± 0.04	0.08 ± 0.03	0.03 ± 0.02	0.01 ± 0.01	0.03 ± 0.02	0.07 ± 0.02
Citronellyl acetate	1331	1656	0.21 ± 0.08	2.33 ± 1.03	0.85 ± 0.30	5.04 ± 1.39	1.15 ± 0.31	0.47 ± 0.13
Geranyl acetate	1357	1751	3.29 ± 0.38	3.45 ± 0.66	2.49 ± 0.39	2.20 ± 0.23	3.44 ± 0.61	2.13 ± 0.28
Methyleugenol	1366	2113	0.07 ± 0.03	0.19 ± 0.08	0.20 ± 0.10	0.27 ± 0.14	0.37 ± 0.10	0.65 ± 0.10
Dodedacanal	1385	1402	–	–	0.01 ± 0.01	–	0.02 ± 0.01	0.03 ± 0.01
(*E*)-*β*-Caryophyllene	1412	1588	8.69 ± 1.60	4.42 ± 1.09	12.54 ± 2.40	19.99 ± 2.32	12.84 ± 0.01	13.22 ± 2.65
2-Undecanyl acetate	1414	1654	–	0.02 ± 0.02	–	–	0.02 ± 0.01	6.18 ± 4.18
(*E*)-Geranylacetone	1425	1847	0.07 ± 0.03	0.23 ± 0.05	0.21 ± 0.04	0.32 ± 0.02	0.30 ± 0.03	0.17 ± 0.02
(*E*)-*α*-Bergamotene	1428	1579	7.13 ± 2.02	4.12 ± 1.21	5.01 ± 2.10	6.61 ± 1.82	6.41 ± 1.53	1.31 ± 0.20
Sesquisabinene	1431	1583	2.51 ± 2.50	–	–	0.01 ± 0.01	0.01 ± 0.01	0.04 ± 0.01
*β*-Barbatene	1434	1635	0.02 ± 0.01	0.05 ± 0.02	0.30 ± 0.07	0.36 ± 0.08	0.21 ± 0.05	0.31 ± 0.06
(*E*)-*β*-Farnesene	1443	1661	0.46 ± 0.20	0.64 ± 0.19	0.10 ± 0.10	0.40 ± 0.20	0.21 ± 0.11	0.25 ± 0.18
*α*-Humulene	1444	1659	1.25 ± 0.42	0.32 ± 0.21	2.58 ± 0.54	4.17 ± 0.47	2.85 ± 0.66	5.31 ± 0.95
Isopentyl-phenylacetate	1458	1988	0.02 ± 0.01	0.18 ± 0.07	0.21 ± 0.05	0.21 ± 0.03	0.30 ± 0.04	0.28 ± 0.06
(*Z*.*Z*)-*α*-Farnesene	1470	1692	0.15 ± 0.06	0.03 ± 0.02	0.11 ± 0.03	0.15 ± 0.04	0.10 ± 0.04	0.38 ± 0.06
(*Z*.*E*)-*α*-Farnesene	1478	1718	0.21 ± 0.06	0.77 ± 0.18	0.35 ± 0.07	0.69 ± 0.06	0.99 ± 0.08	0.39 ± 0.05
*α*-Zingiberene	1482	1691	–	–	–	0.01 ± 0.01	0.02 ± 0.01	0.06 ± 0.01
Cuparene	1488	1725	–	–	0.02 ± 0.01	0.01 ± 0.01	0.01 ± 0.01	0.02 ± 0.01
*β*-Bisabolene	1496	1719	18.26 ± 3.50	2.72 ± 1.00	8.23 ± 1.58	12.03 ± 1.71	6.33 ± 2.02	13.24 ± 1.45
Unidentified compound 1508	1508	1767	12.99 ± 2.50	2.02 ± 0.05	–	–	0.15 ± 0.10	–
*β*-Sesquiphellandrene	1510	1761	–	0.05 ± 0 .77	6.28 ± 1.22	9.53 ± 1.49	5.04 ± 1.73	11.58 ± 1.35
(*E*)-*γ*-Bisabolene	1518	1751	–	–	0.03 ± 0.02	0.04 ± 0.02	0.01 ± 0.01	0.07 ± 0.02
(*Z*)-3-Hexenyl-benzoate	1538	2083	0.02 ± 0.01	–	0.05 ± 0.02	–	0.19 ± 0.01	0.07 ± 0.01
(*E*)-Nerolidol	1543	2033	0.13 ± 0.06	0.28 ± 0.05	0.17 ± 0.06	0.24 ± 0.02	0.40 ± 0.05	0.17 ± 0.03
Caryophyllene-oxide	1563	1966	2.90 ± 0.67	0.85 ± 0.29	4.08 ± 0.85	5.12 ± 0.71	4.27 ± 1.13	5.16 ± 0.70
Germacrenol-b	1568	1977	–	–	0.03 ± 0.02	–	0.02 ± 0.01	0.08 ± 0.02
Fokienol	1584	2180	0.17 ± 0.09	0.03 ± 0.01	0.07 ± 0.03	0.06 ± 0.02	0.04 ± 0.02	0.25 ± 0.03
Tetradecanal	1586	2228	–	–	0.13 ± 0.07	–	0.54 ± 0.17	–
Humulene-epoxide ii	1587	2073	–	0.09 ± 0.04	0.43 ± 0,13	0.54 ± 0.09	–	0.87 ± 0.12
(*Z*)-*α*-Bergamotol	1588	2006	0.30 ± 0.10	0.01 ± 0.01	–	–	0.11 ± 0.06	–
Hexyl-phenylacetate	1595	1975	–	–	0.06 ± 0.04	–	–	–
Caryophylladienol ii	1614	2329	–	–	0.08 ± 0.05	0.01 ± 0.01	0.03 ± 0.02	0.20 ± 0.05
*Delta*-9-10-Eremophilen-11-ol	1623	2178	0.34 ± 0.09	0.29 ± 0.09	0.23 ± 0.05	0.14 ± 0.05	0.24 ± 0.05	0.35 ± 0.05
*α*-Bisabolol	1660	2175	0.63 ± 0.17	0.14 ± 0.05	0.33 ± 0.08	0.41 ± 0.09	0.30 ± 0.07	0.76 ± 0.11
Dodecenyl acetate	1666	2012	0.40 ± 0.12	0.60 ± 0.14	0.38 ± 0.07	0.31 ± 0.09	0.25 ± 0.06	0.34 ± 0.04
Tetradecyl acetate	1787	2369	0.08 ± 0.04	0.17 ± 0.06	0.03 ± 0.02	0.01 ± 0.01	0.10 ± 0.03	0.17 ± 0.06
Heptadecadienone	1824	2297	0.01 ± 0.01	0.01 ± 0.01	0.05 ± 0.01	0.11 ± 0.03	0.27 ± 0.03	0.07 ± 0.03
*α*-Springene	1931	2214	0.46 ± 0.13	1.39 ± 0.29	1.65 ± 0.41	3.01 ± 0.28	1.94 ± 0.26	1.55 ± 0.22
*b*-Springene	1947	2239	0.28 ± 0.09	1.10 ± 0.24	1.14 ± 0.31	2.83 ± 0.27	1.37 ± 0.17	1.21 ± 0.18
Octadecyl acetate	2183	2497	0.04 ± 0.03	0.22 ± 0.07	0.73 ± .0.15	0.90 ± 0.12	0.33 ± 0.06	0.37 ± 0.05

Retention Indices on apolar RI ^b^(ap.) and on polar RI ^c^(p.) columns determined relative to the retention times of a series of *n*-alkanes (C_7_–C_28_) with linear interpolation. An example of the chromatograms analyzed can be found in the Supplementary Materials (see Fig. S1).

The phytochemical analyses (Table 2 – for more information see Table S1 in supplementary materials) showed that the main components detected in the EOs of *T. marum* obtained from the Cagliari population were *β*-bisabolene (18.26%), dolichodial (15.65%), and *(E)*-*β*-caryophyllene (8.69%); from the Alghero population dolichodial (34.38%), 2-methylbutyl-2-methylbutyrate (5.65%), and 2-methylbutyl-butyrate (5.76%); from the Costa Paradiso population *(E)*-*β*-caryophyllene (12.54%), dolichodial (11.25%), and *β*-bisabolene (8.23%); from the Limbara population *(E)*-*β*-caryophyllene (19.99%), *β*-bisabolene (12.03%), and *β*-sesquiphellandrene (9.53%); from the Osilo population *(E)*-*β*-caryophyllene (12.84%), 2-methylbutyl-butyrate (9.54%), and 2-methylbutyl-2-methylbutyrate (7.58%); and finally the principal compounds from the Urzulei population were *β*-bisabolene (13.24%), *(E)*-*β*-caryophyllene (13.22%), and *β*-sesquiphellandrene (11.58%).

The SIMPER analysis (see Supplementary Material Table S2) showed that the volatile compounds listed above, along with four other compounds—namely the unidentified compound 1508 (4.70% contribution to the average population dissimilarity), limonene (7.30%), 2-undecanyl acetate (4.60%), and *(E)*-*α*-bergamotene (3.80%)— were also important in determining population chemical differences. In total, these ten volatile compounds explained 90% of the phytochemical diversity observed in the six studied Sardinian populations. A high percentage of dolichodial was found in the Alghero (34.40%), Cagliari (15.60%), and Costa Paradiso (11.30%) populations, with a much lower quantity found in the population of Limbara (0.50%), and modest quantities in the populations of Urzulei (5.70%) and Osilo (5.30%). In contrast to dolichodial, *(E)*-*β*-caryophyllene was found in higher percentages in the Limbara (20.00%), Urzulei (13.20%), and Osilo (12.80%) populations than those of Costa Paradiso (12.50%), Cagliari (8.70%), or Alghero (4.40%). *β*-bisabolene was found in relatively higher percentages in the Cagliari (18.30%), Urzulei (13.20%), and Limbara (12.00%) populations, in modest quantities in the Costa Paradiso (8.20%) and Osilo (6.30%) populations, and in a lower quantity in Alghero (2.70%). High quantities of unidentified compound 1508 were found only in Cagliari (13.00%), and only traces in the Alghero (2.00%) and Osilo (0.10%) populations. On the other hand, limonene was found only in the Cagliari population (6.20%) as shown in Fig. S2.

The high chemical variability of the *T. marum* EOs, found in the studied Sardinian populations, was also found, in general, in previous studies [5,30,[38], [39], [40],^57^,^58^]. An interesting finding in this research is the presence of dolichodial, a compound of significant economic value, found only in selected populations in the Balearic Islands [39,58], and in a few other populations in Sardinia [5,39], together with other compounds typical of *T. marum*, such as caryophyllene oxide, *α*-bergamotene, *β*-bisabolene, *(E)*-*β*-caryophyllene, *β*-sesquiphellandrene, estragole, geranyl acetate, epidolichodial and *α*-humulene [38]. In other Sardinian [30] and Corsican [40,57] samples, the above-mentioned compound dolichodial has not been found. Still, although this compound is a typical production of the plant, as reported by several studies, it is not always detected in the extraction of EOs [5,58]. Several factors could influence the chemical composition of the EOs, however, regarding the presence or absence of dolichodial in the EO of *T. marum*, an explanation is given by Djabou et al. [40]. Indeed, the Corsican samples’ compound was found, as the main volatile component, in the HS-SPME solid phase extraction process, while it was never detected in the EOs obtained by hydrodistillation (HD). Djabou et al. [40], hypothesised that dolichodial was probably lost by chemical transformation, under thermal conditions and acid pH in hydrodistillation. During the hydrodistillation step, the aldehyde group from the hydrate and the dihydrate forms of the dolichodial was partially transferred to the hydrolated aqueous phase, due to its greater affinity with water. It was found that the presence of dolichodial was in much greater quantities in the Sardinian samples compared with the Corsican samples. This difference may reflect regional variations in its abundance, as a small portion may have been lost during the process. The coastal samples from Cagliari and Alghero had the highest percentage of dolichodial, while the lowest was found in Limbara, and intermediate amounts were found in Osilo and Urzulei. This variation in presence could be linked to altitude and climatic factors, demonstrating the strong influence that environmental factors have on the chemical composition of the EO, as previously reported in previous studies [[1], [2], [3],[14], [15], [16], [17], [18],[20], [21], [22], [23], [24], [25]].

Another important aspect observed was that, in their samples, 2-methylbutyl-2-methylbutyrate and 2-methylbutyl butyrate compounds were the main components in some samples, while they were not detected in other Mediterranean samples. Furthermore, the study of *T. marum* EOs evidenced a similar chemical composition of the main compounds, such as *(E)*-*β*-caryophyllene, *β*-bisabolene, and *β*-sesquiphellandrene, commonly found in other samples analyzed from Sardinia and other Mediterranean islands, such as Corsica and the Balearic Islands [5,30,[38], [39], [40],^57^,^58^].

Variation in secondary-compound composition among individuals, and populations of the same species, is a quite common feature of aromatic Mediterranean vascular plants, and can result from genetic polymorphism in the production of EOs [3,15]. Geographic variation in the composition of EO is well documented, not only in typical aromatic dwarf shrubs like *Salvia rosmarinus* Spenn [35,66]. and *Lavandula latifolia* Medik. [67], but also in evergreen sclerophyllous trees like *Quercus ilex* L [68]. and *Pinus pinaster* Aiton [69]. In the endemic dwarf *Helichrysum italicum* (Roth) G. Don subsp. *tyrrhenicum* (Bacch., Brullo & Giusso) Herrando, J.M. Blanco, L. Sáez & Galbany from Sardinia, geographic variation in EO composition was related not only to population genetic structure, but also to elevation [19,20]. Local environmental factors (like morphology, which influences winter minimum temperatures) explained the phytochemical diversity among *Thymus vulgaris* L. populations, at short spatial scales [70].

### 3.2 Inter- and intra-population chemical variability

**Fig. 2:**
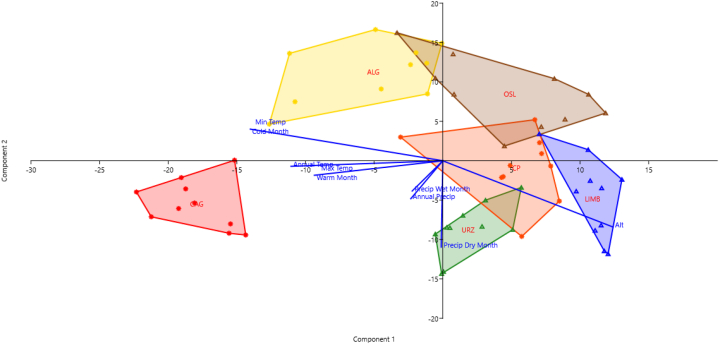
Principal Component Analysis (PCA - axis 1: 16.7%; axis 2: 11.8% of the variance explained) of clr-transformed (Center logratio) chemical composition of EOs of *Teucrium marum* L. from six different natural Sardinian populations, showing the environmental factors as supplementary variables. CAG (Capo S. Elia – Cagliari, CA), ALG (Lazzaretto – Alghero, SS), CP (Costa Paradiso - Trinità d'Agultu e Vignola, SS), LIMB (Limbara - Tempio Pausania, SS), OSL (Osilo, SS), and URZ (Genna Silana – Urzulei, NU).

The EOs obtained from the aerial parts of *T. marum* collected in six different locations in Sardinia displayed varying chemical compositions, as demonstrated in Fig. 2.

The PCA of the EO sample composition (Fig. 2) showed that, overall, all the populations differ from each other, despite the similarity of some individuals belonging to different populations. Indeed, a few overlapping population areas shown on the PCA corresponded to one individual of the Osilo population, which shared the same phytochemical composition with one individual from Alghero, as well as another individual from Osilo that had a similarity in chemical composition with one sample from Costa Paradiso and another from Limbara. Interestingly, the Cagliari and Urzulei populations were the only ones that did not share any individual with the other populations, with the Urzulei population standing out on the third axis of the PCA, higher than all the other populations (figure not shown). The size of the region encompassing a specific population indicates the chemical compositional variability of that population. As it turns out, the populations in Alghero, Osilo, and Costa Paradiso have a greater compositional diversity compared with the populations in Cagliari, Limbara, or Urzulei. The population of Osilo is the most variable, with individuals ‘connecting’ (albeit marginally) the three populations of Alghero, Costa Paradiso, and Limbara. All the other populations have different chemical compositions, with no overlap. The PCA also showed that the distribution of the populations of Cagliari and Alghero was mainly correlated with abiotic factors related to temperature, while the populations of Limbara and Urzulei (and partially Costa Paradiso) were mainly correlated with the precipitation gradient.

The diversity between populations is also confirmed by the non-parametric test ANOSIM, which showed a significant difference in the phytochemical EO composition among the studied populations (ANOSIM: mean rank within: 320.7; mean rank between: 952.4; R = 0.738, *p* = 10^−4^). Moreover, the EO composition differed significantly among all populations according to the multiple pairwise population comparisons (*p* ≤ 0.003, Bonferroni-corrected *p*-values). These population differences in EO composition were significantly explained partly by both the different elevation and the soil substrate (two-way PERMANOVA: elevation F_5,35_ = 4.36, *p* = 0.0001; substrate F_3,35_ = 4.91, *p* = 0.0001; interaction (elevation x soil) F_15,35_ = 0.98, *p* > 0.1).

The dendrogram (see supplementary materials Fig. S3) obtained by Cluster Analysis (CA) showed that, on the basis of their EOs’ chemical composition, the six studied Sardinian populations of *T. marum* were grouped in three main clusters: the first showed major homogeneity and comprised only the Cagliari population, the second showed moderate heterogeneity and included both the Alghero and Osilo populations, with only one individual from the Costa Paradiso population, and the last showed marked heterogeneity and comprised the Limbara, Costa Paradiso, and Urzulei populations.

**Fig. 3:**
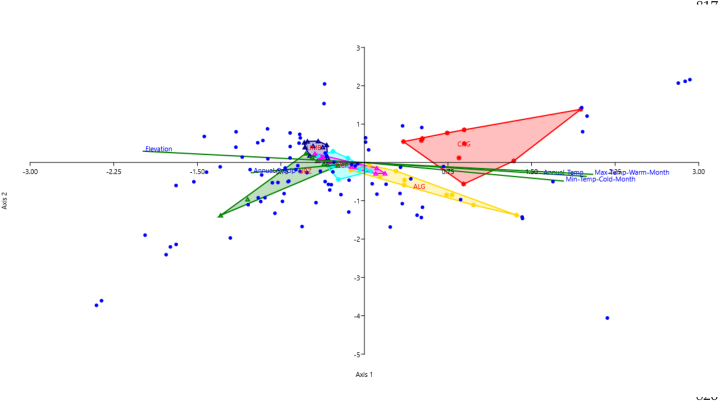
Canonical Correspondence Analysis (CCA - axis 1: 55.7%; axis 2: 22.9% of the variance explained) of chemical composition of EOs of *Teucrium marum* L. from six different natural Sardinian populations, showing the environmental factors as supplementary variables. CAG (Capo S. Elia – Cagliari, CA), ALG (Lazzaretto – Alghero, SS), CP (Costa Paradiso - Trinità d'Agultu e Vignola, SS), LIMB (Limbara - Tempio Pausania, SS), OSL (Osilo, SS), and URZ (Genna Silana – Urzulei, NU). Compounds distributions (see Fig. S2) are marked by blue-points. Environmental variables are represented by vectors; vector length indicates the relative weight of a given variable in the ordination, and direction indicates the correlation of that variable with each axis. Environmental variable mean is placed at the origin; above-average values of a given variable position along its corresponding vector, and below-average values project from the origin in the opposite direction. The variables shown are (1) elevation, (2) annual temperature, (3) maximum temperature of the warmest month, (4) minimum temperature of the coldest month, (5) annual precipitation. (For interpretation of the references to colour in this figure legend, the reader is referred to the Web version of this article.)

The CCA showed the linear combination of compounds with environmental variables (Fig. 3), making up the first two CCA axes, explained 78.50% of the populations data variance (55.70% and 22.90%, respectively). This analysis highlights how elevation and temperature are the major variables that most influence the distribution of compounds in the populations, as well as the distance between populations. Compounds abundance probability are influenced by environmental factors as well as the distribution of the populations. Compounds distributed over mountain populations are negatively correlated with temperature and positively correlated with elevation, on the contrary, coastal populations such as Cagliari and Alghero are positively correlated with temperature and negatively correlated with elevation. A middle ground is found by the Costa Paradiso which is interposed between the two ones, probably due to the same substrate with the Limbara population and due to the slightly high altitude of the other two coastal populations.

The phytochemical diversity among the studied populations of *T. marum* (see Supplementary Material Fig. S3) seems to be biogeographically structured [71]. Each Sardinian population appears to have a significantly different EO composition resulting partly from unique combinations of elevation and soil substrate. Here the elevation is used as a proxy for climatic variables, as they are highly correlated (as shown by PCA and CCA). These environmental factors are probably not the only explanation for the differences in EO composition among populations, because isolation by geographical distance, maybe connected with genetic fragmentation of investigated populations, could play a significant role in determining inter- and intra-population phytochemical diversity. Further studies on characterising the genetic divergence of *T. marum* populations, like those performed on *Thymus* spp [18,23]. or *Helichrysum italicum* (Roth) G. Don [19], would be of great interest to distinguish the role of environmental and genetic factors. The southernmost population from Cagliari is clearly isolated from the others (as is also confirmed, being the only population with limonene and the unidentified compound 1508), since it grows on a fossil island surrounded by alluvial plains mostly unsuitable for the species. The identified phytochemical differences were correlated with edaphic characteristics and geographic distances, suggesting that environmental factors and genetic distances are the main drivers of the phytochemical polymorphism [8,10,11]. The populations from the western side of the island (Alghero and Osilo) are more similar than the eastern populations (Costa Paradiso, Limbara, and Urzulei), even if some individuals do not fit within the population of provenance. Still, the role played by climatic factors in shaping the observed pattern of phytochemical heterogeneity, as highlighted by the PCA (Fig. 2), is more evident when considering the two compounds that mainly characterise the EOs of the studied populations, both of economic interest, dolichodial and (*E*)-*β*-caryophyllene, which follow opposite patterns determined by elevation (and, consequently, by bioclimate). The first compound is much more abundant in coastal populations (Alghero, Cagliari, and Costa Paradiso) than in mountainous ones (Osilo, Limbara, and Urzulei), whereas the second one follows the opposite pattern. Although dolichodial's role in the chemical defence of *T. marum* is well documented [72], and its biosynthesis long understood [73,74], our findings strongly support a significant difference in dolichodial production between the coastal and mountainous populations of cat thyme. Considering the key role of this compound in the anti-insect defence of *T. marum* [72], it is possible that coastal populations produce higher amounts of dolichodial to limit insect damage, in the hottest and driest period of the year (summer). On the other hand, mountain populations growing at higher elevations may be at a lower risk of insect damage during the summer months due to lower temperatures and consequent lower levels of drought stress. Differences in EO composition related to aridity gradients are well documented in another Mediterranean dwarf Lamiaceae, the Tunisian *Thymbra capitata* (L.) Cav [75]. *(E)*-*β*-caryophyllene is a natural sesquiterpene hydrocarbon present in hundreds of plants [76], that is documented to directly inhibit bacterial growth in the model species *Arabidopsis thaliana* (L.) Heynh [77]. It is, therefore, possible that higher amounts of this compound are more useful for populations of *T. marum* growing at higher elevations than for populations living in coastal environments because moister climates generally promote bacterial and fungal development. Therefore, the hypothesis that ecological conditions, such as elevation and soil substrate, have to be considered significant factors in the composition of the EO of the different populations of *T. marum*, and that these can influence the intra- and inter-population phytochemical diversity of the species, cannot be rejected.

## 4 Conclusions

Here for the first time, it was shown that the composition of Sardinian *T. marum* EOs was unique among other Mediterranean known compositions, particularly for the high level of dolichodial found in the coastal populations under study, and that ecological factors play a significant role in shaping the phytochemical diversity among and within wild populations of *Teucrium marum*. In particular, a significant effect of environmental and climatic factors on the EO composition was found, with mountainous populations possessing an EO that is distinct from that of coastal populations. Beyond the ecological factors determining intra- and inter-population phytochemical diversity, the data shown here provide the scientific background to address the sustainable use of wild populations of aromatic plants. The Mediterranean basin is indeed, a hotspot of medicinal and aromatic plants, that have always been used by humans. Recently, however, their exploitation has dramatically increased, and new scenarios are proposed for local development and sustainability, in order to preserve this natural capital [78]. There is, in fact, growing awareness of not only the potential socio-economic benefits, but also the environmental risks determined by the exploitation of wild populations of medicinal and aromatic plants, especially on Mediterranean islands [79,80]. Producing detailed data on the phytochemical characterisation of wild populations of Mediterranean medicinal and aromatic plants, as presented here, is the first step in developing the correct approach to the possible use of natural products for human needs, as already done with other plants like *S. rosmarinus*, used to control the expansion of invasive alien plants [28,29].

The high percentage of dolichodial in the Sardinian coastal populations, compared with the Sardinian mountain populations, and other ones (such as the Corsican samples), is economically remarkable [5,38,81]. To favour the sustainable economic use of *Teucrium marum* EO, simultaneously avoiding genetic erosion due to an unsustainable wild collection [82,83], we strongly suggest Sardinian *T. marum* micropropagation and its multiplication in nursery, to obtain a large number of new individuals [[84], [85], [86]], in accordance with IUCN [87], WWF/TRAFFIC, World Health Organization (WHO), the Convention on Biological Diversity [[88], [89], [90], [91]], and the International Standard on Sustainable Wild Collection of Medicinal and Aromatic Plants [83,92,93] guidelines.

## Author contribution statement

Alfredo Maccioni: Conceived and designed the experiments; Analyzed and interpreted the data; Wrote the paper.

Silvia Macis: Performed the experiments; Analyzed and interpreted the data; Wrote the paper.

Marc Gibernau: Conceived and designed the experiments; Performed the experiments; Analyzed and interpreted the data; Contributed reagents, materials, analysis tools or data; Wrote the paper.

Emmanuele Farris: Conceived and designed the experiments; Analyzed and interpreted the data; Contributed reagents, materials, analysis tools or data; Wrote the paper.

## Data availability statement

Data included in article/supp. material/referenced in article.

## Funding

This research was funded by 10.13039/100014810Fondazione di Sardegna bando 2017 per progetti di ricerca con revisione tra pari, grant name “Plant-plant interactions under and around the canopy of Mediterranean aromatic dwarf shrubs in Corsica and Sardinia”

## Declaration of competing interest

The authors declare that they have no known competing financial interests or personal relationships that could have appeared to influence the work reported in this paper.

## Abbreviations

EO: essential oil
EOs: essential oils
MAPs: Medicinal and Aromatic Plants
CAG: Cagliari population
ALG: Alghero population
CP: Costa Paradiso population
LIMB: Limbara population
URZ: Urzulei population
OSL: Osilo population
GC: Gas Chromatography
MS: Mass Spectrometry
RIs: Retention Indices
Clr: Center logratio
PCA: Principal component Analysis
SIMPER: Similarity Percentage
ANOSIM: Analysis of similarities
PERMANOVA: Permutational Multivariate Analysis of Variance
RI: Retention Index
CCA: Canonical Correspondence Analysis
CA: Cluster Analysis
HD: hydrodistillation
