# Supplementary material for: Phytochemical diversity within and among Sardinian populations of the endemic Teucrium marum L. (Lamiaceae) is determined by ecological factors
Source: Heliyon. 2023 Jun 29;9(7):e17728. doi: 10.1016/j.heliyon.2023.e17728 (PMC10395143; doi:10.1016/j.heliyon.2023.e17728)

**Supplementary Materials**

**Table S1**. Basic descriptive statistics for all samples of univariate data.

| Compounds | No. | Mean | Std. error | Variance | Stand. dev | Median |
| --- | --- | --- | --- | --- | --- | --- |
| 2-methylbutanal | 60 | 0.07 | 0.02 | 0.03 | 0.17 | 0.00 |
| 2-methylbutanol | 60 | 0.09 | 0.01 | 0.01 | 0.11 | 0.04 |
| hexanal | 60 | 0.30 | 0.04 | 0.11 | 0.33 | 0.23 |
| 2-hexenal | 60 | 0.10 | 0.01 | 0.01 | 0.10 | 0.08 |
| hexanol | 60 | 0.11 | 0.02 | 0.02 | 0.14 | 0.09 |
| 2-methylpropyl propionate | 60 | 0.02 | 0.02 | 0.02 | 0.14 | 0.00 |
| 3-methylbutyl acetate | 60 | 0.83 | 0.12 | 0.78 | 0.89 | 0.65 |
| 3-heptenol | 60 | 0.01 | 0.01 | 0.00 | 0.04 | 0.00 |
| 2-methylpropyl butanoate | 60 | 0.24 | 0.04 | 0.09 | 0.30 | 0.14 |
| 3-methylbutyl propanoate | 60 | 0.34 | 0.06 | 0.24 | 0.49 | 0.09 |
| 6-methyl-5-hepten-2-one | 60 | 1.08 | 0.10 | 0.59 | 0.77 | 0.90 |
| butylbutyrate | 60 | 0.02 | 0.01 | 0.00 | 0.04 | 0.00 |
| 2-pentyl furan | 60 | 0.01 | 0.00 | 0.00 | 0.03 | 0.00 |
| myrcene | 60 | 0.02 | 0.01 | 0.01 | 0.09 | 0.00 |
| (*Z*)-3-hexenyl acetate | 60 | 0.04 | 0.01 | 0.00 | 0.05 | 0.03 |
| 2-methyl-propyl-2-methylbutyrate | 60 | 0.14 | 0.03 | 0.04 | 0.20 | 0.05 |
| hexyl acetate | 60 | 1.66 | 0.36 | 7.69 | 2.77 | 0.90 |
| 4-methylanisole | 60 | 0.02 | 0.01 | 0.00 | 0.05 | 0.00 |
| 3-methylbutyl-2-methylpropanoate | 60 | 0.07 | 0.02 | 0.01 | 0.12 | 0.00 |
| limonene | 60 | 0.94 | 0.94 | 51.73 | 7.19 | 0.00 |
| *(E)-β-*ocimene | 60 | 0.10 | 0.05 | 0.17 | 0.41 | 0.00 |
| 2-methylbutyl butyrate | 60 | 4.36 | 0.68 | 27.22 | 5.22 | 2.87 |
| octanol | 60 | 0.02 | 0.00 | 0.00 | 0.03 | 0.00 |
| nonanal | 60 | 0.07 | 0.01 | 0.01 | 0.08 | 0.05 |
| linalool | 60 | 0.76 | 0.10 | 0.60 | 0.77 | 0.54 |
| hexyl propanoate | 60 | 0.58 | 0.12 | 0.85 | 0.92 | 0.22 |
| 2-methylbutyl-2-methylbutyrate | 60 | 3.48 | 0.59 | 20.72 | 4.55 | 1.85 |
| octen-3-yl acetate | 60 | 0.82 | 0.14 | 1.15 | 1.07 | 0.63 |
| *(Z)-*tagetone | 60 | 0.02 | 0.00 | 0.00 | 0.03 | 0.00 |
| citronellal | 60 | 0.91 | 0.14 | 1.11 | 1.05 | 0.68 |
| 1,4-dimethoxy benzene | 60 | 0.14 | 0.02 | 0.02 | 0.15 | 0.10 |
| 3-methylbutyl pentanoate | 60 | 0.04 | 0.01 | 0.00 | 0.07 | 0.00 |
| nonanol | 60 | 0.01 | 0.00 | 0.00 | 0.02 | 0.00 |
| (*E*)-3-hexenyl butyrate | 60 | 0.07 | 0.01 | 0.01 | 0.08 | 0.06 |
| *α*-terpineol | 60 | 0.02 | 0.01 | 0.00 | 0.05 | 0.00 |
| estragole hexyl butyrate | 60 | 4.66 | 0.37 | 8.04 | 2.83 | 4.36 |
| isopentyl-hexanoate | 60 | 0.04 | 0.01 | 0.00 | 0.07 | 0.00 |
| 3,4-dimethoxytoluene | 60 | 0.12 | 0.03 | 0.05 | 0.23 | 0.00 |
| citronellol | 60 | 0.16 | 0.04 | 0.09 | 0.30 | 0.00 |
| neral | 60 | 0.18 | 0.04 | 0.10 | 0.32 | 0.05 |
| (*Z*)-3-hexenyl-2-methylbutanoate | 60 | 0.05 | 0.01 | 0.01 | 0.07 | 0.00 |
| hexyl 2-methylbutanoate | 60 | 1.02 | 0.16 | 1.47 | 1.21 | 0.56 |
| phenylethyl acetate | 60 | 0.36 | 0.05 | 0.14 | 0.37 | 0.26 |
| geraniol | 60 | 0.17 | 0.04 | 0.09 | 0.30 | 0.06 |
| 2-methylbutyl hexanoate | 60 | 0.16 | 0.03 | 0.05 | 0.22 | 0.06 |
| geranial | 60 | 0.47 | 0.08 | 0.40 | 0.63 | 0.21 |
| dolichodial | 60 | 12.09 | 2.11 | 263.70 | 16.24 | 7.54 |
| epidolichodial | 60 | 1.72 | 0.32 | 5.98 | 2.45 | 0.70 |
| 2-undecanone | 60 | 0.17 | 0.02 | 0.03 | 0.18 | 0.15 |
| dihydroedulan | 60 | 0.12 | 0.01 | 0.01 | 0.11 | 0.11 |
| 6-undecanol | 60 | 0.01 | 0.00 | 0.00 | 0.02 | 0.00 |
| benzyl butyrate | 60 | 0.01 | 0.00 | 0.00 | 0.03 | 0.00 |
| eugenol | 60 | 0.05 | 0.01 | 0.01 | 0.09 | 0.00 |
| citronellyl acetate | 60 | 1.70 | 0.36 | 7.73 | 2.78 | 0.65 |
| geranyl acetate | 60 | 2.82 | 0.19 | 2.20 | 1.48 | 2.56 |
| methyleugenol | 60 | 0.30 | 0.05 | 0.12 | 0.35 | 0.16 |
| dodedacanal | 60 | 0.01 | 0.00 | 0.00 | 0.03 | 0.00 |
| *(E)-β-*caryophyllene | 60 | 12.01 | 1.06 | 66.77 | 8.17 | 11.33 |
| 2-undecanyl acetate | 60 | 1.06 | 0.74 | 32.61 | 5.71 | 0.00 |
| (*E*)-geranylacetone | 60 | 0.22 | 0.02 | 0.02 | 0.13 | 0.22 |
| (*E*)-*α*-bergamotene | 60 | 5.07 | 0.68 | 26.95 | 5.19 | 3.04 |
| sesquisabinene | 60 | 0.39 | 0.38 | 8.61 | 2.93 | 0.00 |
| *β*-barbatene | 60 | 0.21 | 0.03 | 0.05 | 0.21 | 0.16 |
| (*E*)-*β*-farnesene | 60 | 0.34 | 0.07 | 0.29 | 0.54 | 0.00 |
| *α*-humulene | 60 | 2.77 | 0.32 | 6.02 | 2.45 | 2.63 |
| isopentyl phenylacetate | 60 | 0.20 | 0.02 | 0.03 | 0.17 | 0.20 |
| (*Z,Z*)-*α*-farnesene | 60 | 0.15 | 0.02 | 0.03 | 0.17 | 0.09 |
| (*Z*,*E*)-*α*-farnesene | 60 | 0.57 | 0.05 | 0.15 | 0.39 | 0.54 |
| *α*-zingiberene | 60 | 0.01 | 0.00 | 0.00 | 0.03 | 0.00 |
| cuparene | 60 | 0.01 | 0.00 | 0.00 | 0.02 | 0.00 |
| *β*-bisabolene | 60 | 10.00 | 1.01 | 59.83 | 7.73 | 9.38 |
| Cpd 1508 | 60 | 2.02 | 0.71 | 29.87 | 5.47 | 0.00 |
| *β*-sesquiphellandrene | 60 | 5.84 | 0.71 | 30.09 | 5.49 | 5.39 |
| (*E*)-*γ*-bisabolene | 60 | 0.03 | 0.01 | 0.00 | 0.05 | 0.00 |
| (*Z*)-3*-*hexenyl-benzoate | 60 | 0.05 | 0.01 | 0.01 | 0.08 | 0.00 |
| (*E*)-nerolidol | 60 | 0.23 | 0.02 | 0.03 | 0.16 | 0.23 |
| caryophyllene oxide | 60 | 3.74 | 0.36 | 7.63 | 2.76 | 3.40 |
| germacrenol b | 60 | 0.02 | 0.01 | 0.00 | 0.05 | 0.00 |
| fokienol | 60 | 0.10 | 0.02 | 0.02 | 0.14 | 0.07 |
| tetradecanal | 60 | 0.11 | 0.04 | 0.09 | 0.30 | 0.00 |
| humulene epoxide ii | 60 | 0.33 | 0.05 | 0.17 | 0.41 | 0.12 |
| (*Z*)-*α*-bergamotol | 60 | 0.07 | 0.02 | 0.03 | 0.17 | 0.00 |
| hexyl phenylacetate | 60 | 0.01 | 0.01 | 0.00 | 0.05 | 0.00 |
| caryophylladienol ii | 60 | 0.06 | 0.02 | 0.01 | 0.12 | 0.00 |
| *Delta*-9,10-eremophilen-11-ol | 60 | 0.26 | 0.03 | 0.04 | 0.21 | 0.23 |
| *α*-bisabolol | 60 | 0.42 | 0.05 | 0.13 | 0.37 | 0.31 |
| dodecenyl acetate | 60 | 0.38 | 0.04 | 0.09 | 0.30 | 0.31 |
| tetradecyl acetate | 60 | 0.09 | 0.02 | 0.02 | 0.13 | 0.05 |
| heptadecadienone | 60 | 0.09 | 0.01 | 0.01 | 0.11 | 0.05 |
| *α*-springene | 60 | 1.69 | 0.15 | 1.27 | 1.13 | 1.76 |
| *b*-springene | 60 | 1.34 | 0.13 | 1.04 | 1.02 | 1.14 |
| octadecyl acetate | 60 | 0.44 | 0.05 | 0.16 | 0.40 | 0.40 |

No.: is the number of values *n* in the sample (60 individuals of *Teucrium marum* L.); Mean: is the estimate of the mean, calculated as $x ̅=(\sum x\_i)/n$ ; Std. error: is the standard error of the estimate of the mean, calculated as $SEx ̅=s/\surd n$ where s is the estimate of the standard deviation; Variance: is the sample variance, calculated as $s^2=1/(n-1)\sum(x\_i-x ̅)^2$; Stand. dev.: is the sample standard deviation, calculated as $s=\surd(1/(n-1)\sum(x\_i-x ̅)^2 )$; Median: is the median of the sample: for *n* odd $\mathrm{median}(x)=x\_((n+1)/2)$ *;* for *n* even $\mathrm{median}(x)=(x\_((n/2) )+x\_(((n/2)+1)))/2$ .

**Table S2.** The SIMPER Analysis (with no data transformation) with Euclidean distance, showing the differences in chemical composition among different Sardinian wild populations of *Teucrium marum* L. CAG (Capo S. Elia – Cagliari, CA), ALG (Lazzaretto – Alghero, SS), CP (Costa Paradiso - Trinità d'Agultu e Vignola, SS), LIMB (Limbara - Tempio Pausania, SS), OSL (Osilo, SS) and URZ (Genna Silana – Urzulei, NU).

| Compound | Av. Dissim | Contrib. % | Cumulative % | Mean LIMB  % | Mean CAG  % | Mean ALG  % | Mean URZ  % | Mean CP  % | Mean OSL  % |
| --- | --- | --- | --- | --- | --- | --- | --- | --- | --- |
| dolichodial | 568.10 | 39.59 | 39.59 | 0.51 | 15.60 | 34.40 | 5.77 | 11.3 | 5.32 |
| (*E*)-*β*-caryophyllene | 140.20 | 9.77 | 49.36 | 20.00 | 8.69 | 4.42 | 13.2 | 12.5 | 12.8 |
| *β*-bisabolene | 127.90 | 8.91 | 58.27 | 12.00 | 18.30 | 2.72 | 13.20 | 8.23 | 6.33 |
| limonene | 105.30 | 7.33 | 65.61 | 0.00 | 6.16 | 0.00 | 0.00 | 0.00 | 0.00 |
| Cpd 1508 | 67.69 | 4.72 | 70.33 | 0.00 | 13.00 | 0.05 | 0.00 | 0.00 | 0.15 |
| 2-undecanyl acetate | 66.09 | 4.60 | 74.93 | 0.00 | 0.00 | 0.02 | 6.18 | 0.00 | 0.02 |
| *β*-sesquiphellandrene | 65.37 | 4.55 | 79.49 | 9.53 | 0.00 | 2.02 | 11.6 | 6.28 | 5.04 |
| 2-methylbutyl butyrate | 57.73 | 4.02 | 83.51 | 2.55 | 0.04 | 5.76 | 1.26 | 6.56 | 9.54 |
| (*E*)-*α*-bergamotene | 54.62 | 3.81 | 87.31 | 6.61 | 7.13 | 4.12 | 1.31 | 5.01 | 6.41 |
| 2-methylbutyl-2-methylbutyrate | 44.02 | 3.07 | 90.38 | 1.29 | 0.00 | 5.94 | 0.61 | 5.09 | 7.58 |
| sesquisabinene | 17.52 | 1.22 | 91.60 | 0.01 | 2.51 | 0.00 | 0.04 | 0.00 | 0.01 |
| estragole + hexylbutyrate | 16.71 | 1.16 | 92.77 | 5.45 | 1.19 | 4.51 | 5.40 | 5.92 | 5.15 |
| citronellyl acetate | 16.25 | 1.13 | 93.9 | 5.04 | 0.21 | 2.33 | 0.47 | 0.85 | 1.15 |
| caryophyllene oxide | 15.90 | 1.11 | 95.01 | 5.12 | 2.90 | 0.84 | 5.16 | 4.08 | 4.27 |
| hexyl acetate | 15.85 | 1.10 | 96.11 | 1.66 | 0.35 | 1.19 | 1.52 | 4.54 | 0.59 |
| *α*-humulene | 12.95 | 0.90 | 97.02 | 4.17 | 1.25 | 0.32 | 5.31 | 2.58 | 2.85 |
| epidolichodial | 12.77 | 0.89 | 97.90 | 0.02 | 2.54 | 4.76 | 1.12 | 1.15 | 0.82 |
| geranyl acetate | 4.46 | 0.31 | 98.22 | 2.20 | 3.29 | 3.45 | 2.13 | 2.49 | 3.44 |
| hexyl-2-methylbutanoate | 3.09 | 0.21 | 98.43 | 1.60 | 0.00 | 0.33 | 0.86 | 2.18 | 1.03 |
| *α*-springene | 2.72 | 0.19 | 98.62 | 3.01 | 0.457 | 1.40 | 1.55 | 1.65 | 1.94 |
| octen-3-y acetate | 2.38 | 0.16 | 98.79 | 0.86 | 0.36 | 0.35 | 1.11 | 1.81 | 0.36 |
| *b*-springene | 2.27 | 0.16 | 98.94 | 2.83 | 0.28 | 1.10 | 1.21 | 1.14 | 1.37 |
| citronellal | 2.26 | 0.16 | 99.10 | 1.12 | 1.73 | 0.70 | 0.49 | 0.39 | 1.14 |
| hexyl propanoate | 1.76 | 0.12 | 99.23 | 0.89 | 0.00 | 0.40 | 0.11 | 1.45 | 0.57 |
| 3-methylbutyl acetate | 1.59 | 0.11 | 99.34 | 0.21 | 0.59 | 1.00 | 1.36 | 1.01 | 0.78 |
| linalool | 1.32 | 0.09 | 99.43 | 0.17 | 0.40 | 0.53 | 0.71 | 0.62 | 2.07 |
| 6-methyl-5-hepten-2-one | 1.21 | 0.08 | 99.51 | 0.95 | 1.15 | 0.82 | 1.14 | 1.73 | 0.68 |
| geranial | 0.83 | 0.06 | 99.57 | 0.15 | 0.45 | 0.29 | 0.99 | 0.15 | 0.75 |
| (*E*)-*β*-farnesene | 0.58 | 0.04 | 99.61 | 0.40 | 0.46 | 0.64 | 0.25 | 0.10 | 0.21 |
| 3-methylbutyl propanoate | 0.52 | 0.03 | 99.65 | 0.18 | 0.00 | 0.72 | 0.04 | 0.19 | 0.89 |
| humulene epoxide ii | 0.37 | 0.02 | 99.67 | 0.54 | 0.00 | 0.09 | 0.87 | 0.43 | 0.00 |
| octadecyl acetate | 0.35 | 0.02 | 99.70 | 0.90 | 0.04 | 0.22 | 0.37 | 0.73 | 0.33 |
| (*E*)-*β*-ocimene | 0.35 | 0.02 | 99.72 | 0.02 | 0.45 | 0.06 | 0.01 | 0.02 | 0.09 |
| (*Z*,*E*)-*α*-farnesene | 0.33 | 0.02 | 99.75 | 0.69 | 0.21 | 0.77 | 0.39 | 0.35 | 0.98 |
| phenylethyl acetate | 0.30 | 0.02 | 99.77 | 0.13 | 0.18 | 0.38 | 0.86 | 0.45 | 0.11 |
| *α*-bisabolol | 0.28 | 0.02 | 99.79 | 0.41 | 0.62 | 0.14 | 0.76 | 0.33 | 0.30 |
| methyleugenol | 0.25 | 0.02 | 99.80 | 0.27 | 0.07 | 0.19 | 0.65 | 0.20 | 0.37 |
| hexanal | 0.23 | 0.01 | 99.82 | 0.26 | 0.12 | 0.13 | 0.29 | 0.72 | 0.23 |
| neral | 0.21 | 0.01 | 99.83 | 0.04 | 0.00 | 0.12 | 0.52 | 0.06 | 0.34 |
| tetradecanal | 0.20 | 0.01 | 99.85 | 0.00 | 0.00 | 0.00 | 0.00 | 0.13 | 0.53 |
| geraniol | 0.20 | 0.01 | 99.86 | 0.02 | 0.03 | 0.05 | 0.15 | 0.03 | 0.70 |
| 2-methylpropyl-butanoate | 0.19 | 0.01 | 99.88 | 0.15 | 0.00 | 0.40 | 0.04 | 0.19 | 0.62 |
| citronellol | 0.18 | 0.01 | 99.89 | 0.40 | 0.17 | 0.04 | 0.08 | 0.01 | 0.24 |
| dodecenyl acetate | 0.18 | 0.01 | 99.90 | 0.31 | 0.40 | 0.60 | 0.34 | 0.38 | 0.25 |
| 3,4-dimethoxytoluene | 0.11 | 0.01 | 99.91 | 0.02 | 0.20 | 0.20 | 0.14 | 0.02 | 0.13 |
| 2-methylbutyl-hexanoate | 0.11 | 0.01 | 99.92 | 0.07 | 0.01 | 0.09 | 0.28 | 0.07 | 0.44 |
| *β*-barbatene | 0.09 | 0.01 | 99.92 | 0.36 | 0.02 | 0.05 | 0.31 | 0.30 | 0.20 |
| 2-methyl-propyl-2-methylbutyrate | 0.09 | 0.01 | 99.93 | 0.05 | 0.00 | 0.30 | 0.02 | 0.12 | 0.33 |
| *Delta*-9,10-eremophilen-11-ol | 0.09 | 0.01 | 99.93 | 0.14 | 0.34 | 0.29 | 0.35 | 0.23 | 0.24 |
| 2-undecanone | 0.07 | 0.00 | 99.94 | 0.05 | 0.20 | 0.07 | 0.31 | 0.22 | 0.20 |
| (*Z*,*Z*)-*α*-farnesene | 0.06 | 0.00 | 99.94 | 0.15 | 0.15 | 0.03 | 0.38 | 0.11 | 0.10 |
| (*Z*)-*α*-bergamotol | 0.06 | 0.00 | 99.95 | 0.00 | 0.30 | 0.01 | 0.00 | 0.00 | 0.11 |
| 2-methylbutanal | 0.06 | 0.00 | 99.95 | 0.00 | 0.24 | 0.01 | 0.08 | 0.02 | 0.05 |
| isopentyl phenylacetate | 0.06 | 0.00 | 99.96 | 0.21 | 0.02 | 0.18 | 0.28 | 0.21 | 0.30 |
| (*E*)-nerolidol | 0.05 | 0.00 | 99.96 | 0.24 | 0.13 | 0.28 | 0.16 | 0.17 | 0.40 |
| 1,4-dimethoxy benzene | 0.04 | 0.00 | 99.96 | 0.19 | 0.08 | 0.17 | 0.06 | 0.21 | 0.10 |
| fokienol | 0.04 | 0.00 | 99.97 | 0.06 | 0.17 | 0.03 | 0.25 | 0.07 | 0.04 |
| 2-methylpropyl propionate | 0.04 | 0.00 | 99.97 | 0.00 | 0.00 | 0.15 | 0.00 | 0.00 | 0.00 |
| hexanol | 0.04 | 0.00 | 99.97 | 0.08 | 0.02 | 0.00 | 0.14 | 0.25 | 0.17 |
| (*E*)-geranylacetone | 0.04 | 0.00 | 99.98 | 0.33 | 0.07 | 0.23 | 0.17 | 0.21 | 0.30 |
| tetradecyl acetate | 0.03 | 0.00 | 99.98 | 0.01 | 0.08 | 0.17 | 0.17 | 0.03 | 0.10 |
| 3-methylbutyl-2-methylpropanoate | 0.03 | 0.00 | 99.98 | 0.02 | 0.00 | 0.18 | 0.00 | 0.05 | 0.19 |
| caryophylladienol ii | 0.03 | 0.00 | 99.98 | 0.01 | 0.00 | 0.00 | 0.20 | 0.08 | 0.03 |
| heptadecadienone | 0.03 | 0.00 | 99.98 | 0.11 | 0.01 | 0.01 | 0.06 | 0.05 | 0.27 |
| 2-methylbutanol | 0.03 | 0.00 | 99.99 | 0.00 | 0.04 | 0.07 | 0.08 | 0.06 | 0.25 |
| dihydroedulan | 0.02 | 0.00 | 99.99 | 0.10 | 0.11 | 0.05 | 0.15 | 0.07 | 0.23 |
| 2-hexenal | 0.02 | 0.00 | 99.99 | 0.16 | 0.07 | 0.03 | 0.21 | 0.01 | 0.14 |
| myrcene | 0.01 | 0.00 | 99.99 | 0.00 | 0.07 | 0.01 | 0.00 | 0.00 | 0.02 |
| (*E*)-3-hexenyl butyrate | 0.01 | 0.00 | 99.99 | 0.09 | 0.00 | 0.05 | 0.11 | 0.01 | 0.15 |
| eugenol | 0.01 | 0.00 | 99.99 | 0.01 | 0.06 | 0.08 | 0.07 | 0.03 | 0.03 |
| nonanal | 0.01 | 0.00 | 99.99 | 0.11 | 0.00 | 0.00 | 0.07 | 0.02 | 0.18 |
| (*Z*)-3-hexenyl-benzoate | 0.01 | 0.00 | 99.99 | 0.00 | 0.02 | 0.00 | 0.06 | 0.05 | 0.19 |
| (*Z*)-3-hexenyl-2-methylbutanoate | 0.01 | 0.00 | 99.99 | 0.02 | 0.00 | 0.04 | 0.07 | 0.02 | 0.13 |
| 3-methylbutyl pentanoate | 0.01 | 0.00 | 100 | 0.01 | 0.00 | 0.03 | 0.01 | 0.02 | 0.14 |
| isopentyl hexanoate | 0.01 | 0.00 | 100 | 0.00 | 0.04 | 0.00 | 0.05 | 0.00 | 0.14 |
| *α*-terpineol | 0.01 | 0.00 | 100 | 0.00 | 0.00 | 0.01 | 0.03 | 0.00 | 0.09 |
| hexyl phenylacetate | 0.01 | 0.00 | 100 | 0.00 | 0.00 | 0.00 | 0.00 | 0.06 | 0.00 |
| (*Z*)-3-hexenyl acetate | 0.01 | 0.00 | 100 | 0.05 | 0.00 | 0.06 | 0.10 | 0.01 | 0.04 |
| 4-methylanisole | 0.01 | 0.00 | 100 | 0.02 | 0.04 | 0.06 | 0.00 | 0.01 | 0.00 |
| (*E*)-*γ*-bisabolene | 0.01 | 0.00 | 100 | 0.04 | 0.00 | 0.00 | 0.07 | 0.03 | 0.01 |
| germacrenol b | 0.00 | 0.00 | 100 | 0.00 | 0.00 | 0.00 | 0.08 | 0.03 | 0.02 |
| 3-heptenol | 0.00 | 0.00 | 100 | 0.00 | 0.00 | 0.00 | 0.07 | 0.00 | 0.00 |
| butyl butyrate | 0.00 | 0.00 | 100 | 0.02 | 0.00 | 0.02 | 0.00 | 0.03 | 0.04 |
| *α*-zingiberene | 0.00 | 0.00 | 100 | 0.01 | 0.00 | 0.00 | 0.06 | 0.00 | 0.02 |
| (*Z*)-tagetone | 0.00 | 0.00 | 100 | 0.01 | 0.00 | 0.01 | 0.03 | 0.00 | 0.05 |
| 2-pentyl furan | 0.00 | 0.00 | 100 | 0.02 | 0.00 | 0.00 | 0.02 | 0.00 | 0.03 |
| dodedacanal | 0.00 | 0.00 | 100 | 0.00 | 0.00 | 0.00 | 0.03 | 0.01 | 0.02 |
| octanol | 0.00 | 0.00 | 100 | 0.01 | 0.03 | 0.00 | 0.02 | 0.01 | 0.02 |
| benzyl butyrate | 0.00 | 0.00 | 100 | 0.01 | 0.00 | 0.03 | 0.00 | 0.00 | 0.02 |
| cuparene | 0.00 | 0.00 | 100 | 0.01 | 0.00 | 0.00 | 0.02 | 0.02 | 0.01 |
| nonanol | 0.00 | 0.00 | 100 | 0.01 | 0.00 | 0.01 | 0.01 | 0.01 | 0.02 |
| 6-undecanol | 0.00 | 0.00 | 100 | 0.00 | 0.01 | 0.00 | 0.01 | 0.00 | 0.02 |

**Figure S1.** Chromatograms relative to the individual CAG-04: a) apolar; b) polar. The chromatogram (examined with AMDIS software) highlights the highest peaks of the compounds dolichodial and (*E*)-*α*-bergamotene. Pentane was not considered for the purposes of the analyses.


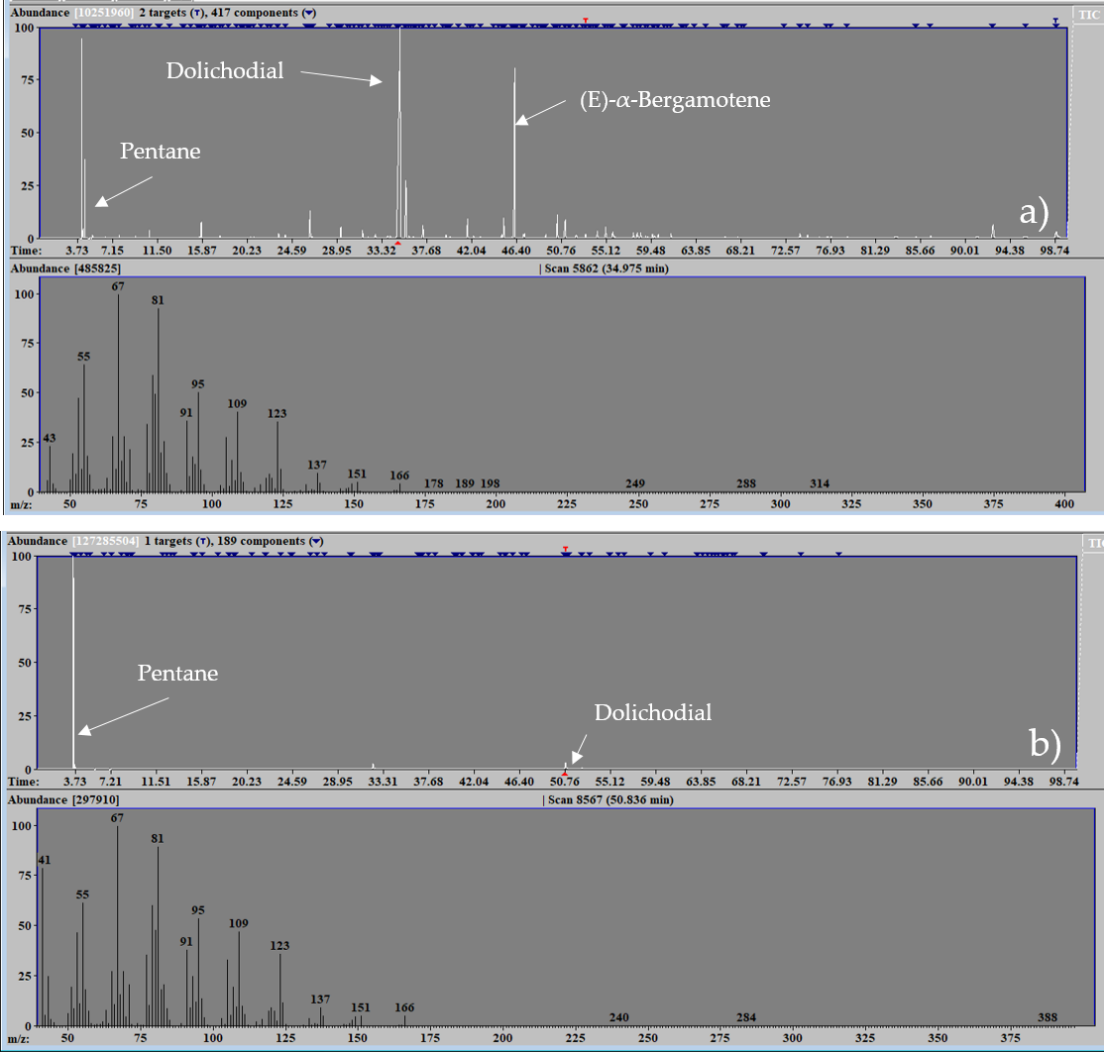


**Figure S2.** Canonical Correspondence Analysis (CCA - axis 1: 55.7%; axis 2: 22.9% of the variance explained) of chemical composition of EOs of Teucrium marum L. from six different natural Sardinian populations, showing the environmental factors as supplementary variables. CAG (Capo S. Elia – Cagliari, CA), ALG (Lazzaretto – Alghero, SS), CP (Costa Paradiso - Trinità d'Agultu e Vignola, SS), LIMB (Limbara - Tempio Pausania, SS), OSL (Osilo, SS), and URZ (Genna Silana – Urzulei, NU). Compounds distributions (see Figure S2) are marked by blue-points. Environmental variables are represented by vectors; vector length indicates the relative weight of a given variable in the ordination, and direction indicates the correlation of that variable with each axis. Environmental variable mean is placed at the origin; above-average values of a given variable position along its corresponding vector, and below-average values project from the origin in the opposite direction. The variables shown are (1) elevation, (2) annual temperature, (3) maximum temperature of the warmest month, (4) minimum temperature of the coldest month, (5) annual precipitation.


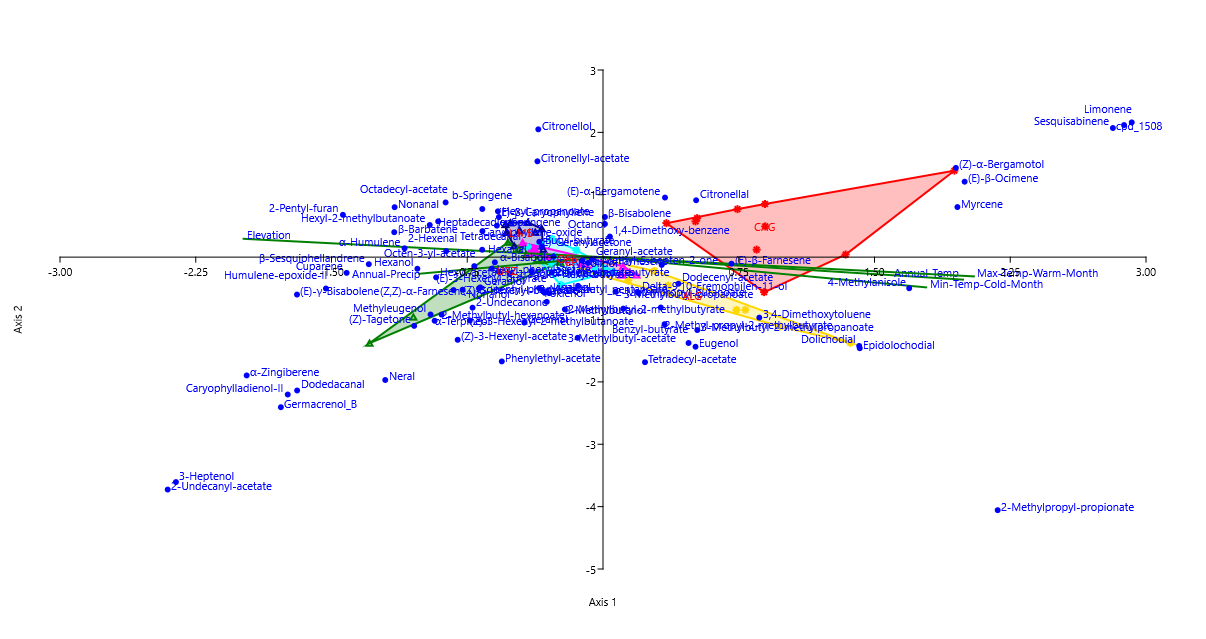


**Figure S3.** Cluster analysis (dendrogram Ward’s method with altitude and substrate clr-transformed) showing the differences in chemical composition among different Sardinian wild populations of *Teucrium marum* L.: CAG (Capo S. Elia – Cagliari, CA), ALG (Lazzaretto – Alghero, SS), CP (Costa Paradiso - Trinità d'Agultu e Vignola, SS), LIMB (Limbara - Tempio Pausania, SS), OSL (Osilo, SS), and URZ (Genna Silana – Urzulei, NU).


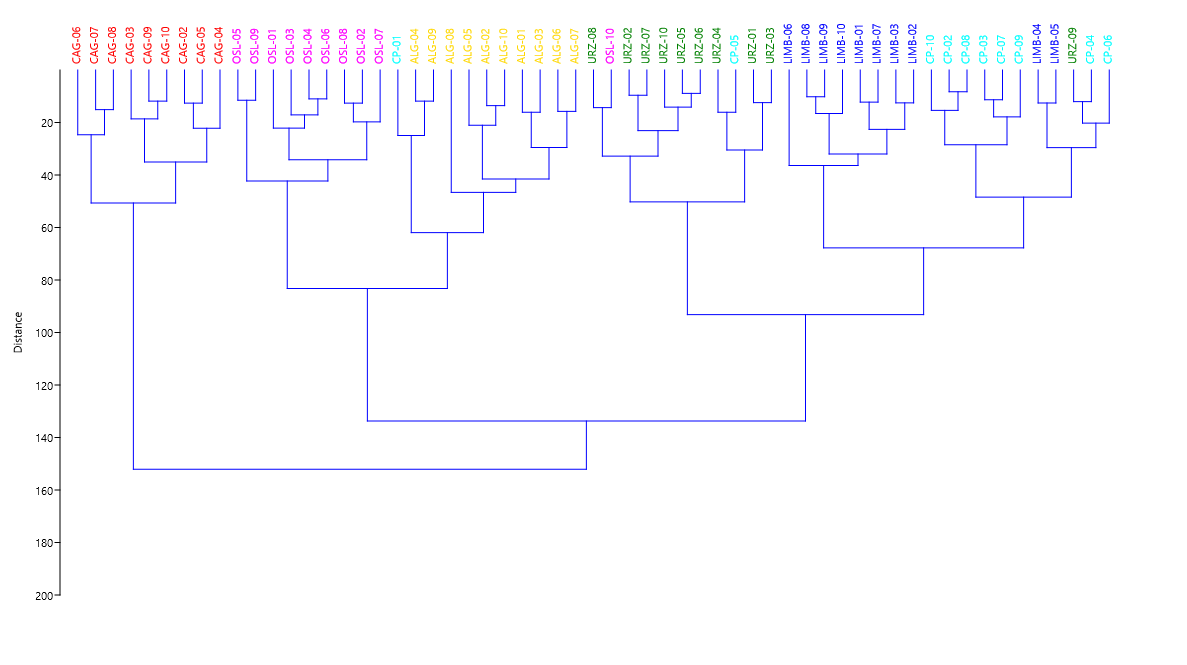

Supplement: Multimedia component 1 [file mmc1.docx]
